# Investigation of retinal microcirculation alterations following carotid artery angioplasty and stenting using optical coherence tomography angiography

**DOI:** 10.3389/fnins.2025.1557062

**Published:** 2025-03-19

**Authors:** Zehui Shi, Chunqiong Dong, Hanfei Tang, Daqiao Guo, Xianglian Li, Bing Xie, Xiao Tang, Xiuping Chen

**Affiliations:** ^1^Department of Ophthalmology, Zhongshan Hospital of Fudan University, Shanghai, China; ^2^Department of Vascular Surgery, Zhongshan Hospital of Fudan University, Shanghai, China; ^3^Institute of Vascular Surgery, Fudan University, Shanghai, China; ^4^Department of Ophthalmology, Ruijin Hospital, Shanghai JiaoTong University School of Medicine, Shanghai, China

**Keywords:** optical coherence tomography angiography, carotid artery stenosis, carotid artery angioplasty and stenting, vascular density, retinal microcirculation

## Abstract

**Background:**

Carotid artery stenosis (CAS) is a common vascular condition that can impair retinal and optic nerve blood supply, leading to ocular ischemic damage. Optical coherence tomography angiography (OCTA) provides a non-invasive method to evaluate retinal microcirculation and detect vascular changes in CAS patients.

**Methods:**

This study utilized OCTA to evaluate changes in retinal microcirculation in CAS patients before and after carotid artery angioplasty and stenting. A 6 × 6 mm OCTA scan was performed to analyze deep retinal vascular complex (DVC) blood flow density, foveal avascular zone area (FAZA), foveal density within a 300 μm-wide ring surrounding the FAZ (FD-300), and radial peripapillary capillary vessel density (RPC-VD). Additionally, ultra-wide-field OCTA imaging (24 × 20 mm) was employed to comprehensively assess perfusion in both the posterior pole and peripheral retina.

**Results:**

Following carotid artery angioplasty and stenting, significant improvements were observed in the ipsilateral eye, including increased macular blood flow density (*p* = 0.004), FD-300 (*p* = 0.031), RPC-VD (*p* = 0.028) and decreased FAZA (*p* = 0.018) in the deep retinal vascular layer, indicating enhanced retinal microcirculation. No statistically significant changes were found in the contralateral eye. In some cases, ultra-wide-field OCTA revealed a reduction in macular non-perfusion areas in the ipsilateral eye, alongside an increase in non-perfusion areas near the vascular arcades.

**Conclusion:**

Carotid artery angioplasty and stenting effectively improves retinal microcirculation in CAS patients, as evidenced by increased blood flow density and reduced non-perfusion areas in the ipsilateral eye. OCTA is a valuable non-invasive tool for assessing retinal circulation dynamics, identifying microvascular abnormalities, and monitoring treatment efficacy in CAS patients.

## Introduction

Carotid artery stenosis (CAS) is a leading cause of stroke (Bonati et al., 2022), affecting approximately 5–10% of individuals aged 70 and older, with luminal narrowing reaching 50% or greater in these cases (de Weerd et al., 2009; Dharmakidari et al., 2017). Around 90% of CAS cases result from atherosclerosis, influenced by hemodynamic, genetic, and metabolic factors at the carotid bifurcation. These factors contribute to endothelial injury, lipid deposition, and the development of fatty streaks, which gradually evolve into atherosclerotic plaque formation, progressively narrowing the artery lumen (Safian, 2017). The continued progression of CAS or sudden rupture of plaques can result in thrombus formation or emboli dislodgment, triggering transient ischemic attacks (TIA) or stroke. Multiple large-scale, multicenter randomized trials have demonstrated that effective management of risk factors, coupled with antiplatelet therapy and surgical interventions such as carotid endarterectomy or carotid angioplasty and stenting, significantly reduces the risk of ischemic cerebrovascular events in patients with moderate to severe symptomatic CAS and selected cases of asymptomatic CAS (Featherstone et al., 2016; Reiff et al., 2022). However, treatment selection for asymptomatic or atypically symptomatic CAS patients remains controversial (Kim et al., 2023). This underscores the necessity for a simple, reproducible non-neurological marker that can reliably reflect hemodynamic changes and can predict adverse outcomes in CAS patients.

The retinal vasculature, which can be directly observed *in vivo*, serves as a reflection of the systemic circulatory status. As the ophthalmic arteries are the first branches of the internal carotid artery, stenosis or occlusive lesions in the common carotid artery (CCA) or internal carotid artery (ICA) may lead to ocular hypoperfusion, manifesting as transient visual disturbances or vision loss-symptoms that often precede cerebrovascular events such as ischemic stroke (Avery et al., 2019; István et al., 2021). Numerous studies have proposed that abnormal retinal microvascular characteristics could serve as novel biomarkers for assessing the severity of cardiovascular disease, neurodegenerative conditions, and microvascular disorders (Baker et al., 2008; Cabrera DeBuc et al., 2017; Dumitrascu et al., 2018). This is largely attributed to the retina’s high metabolic activity, particularly in photoreceptor cells, and its sensitivity to hypoxia (Stefánsson et al., 2019). Ocular ischemic syndrome (OIS), a severe ocular complication associated with CAS, occurs from chronic ocular hypoperfusion caused by >90% stenosis of the CCA or ICA (Lee et al., 2022). Most OIS patients already present with significant visual decline at diagnosis, often accompanied by complications like retinal vein occlusion or neovascular glaucoma, with a generally poor prognosis (Kim et al., 2017; Terelak-Borys et al., 2012). However, chronic CAS does not always exhibit ocular symptoms or detectable retinal structural or functional abnormalities. Traditional diagnostic methods, such as ophthalmoscopy and fundus photography, are often insufficient for detailed assessment of the retinal vasculature, while fluorescein fundus angiography (FFA), though more comprehensive, is invasive and carries risks of allergic reactions, making it unsuitable for routine clinical use. Consequently, our understanding of retinal circulation in these CAS patients remains limited.

Optical coherence tomography angiography (OCTA) is an emerging non-invasive imaging technology that provides high-resolution visualization of the retinal microvascular across various vascular layers with depth-resolving capabilities, allowing for quantitative analysis of vascular parameters such as the foveal avascular zone (FAZ) and vascular density (VD; Spaide et al., 2018). OCTA has been widely applied in the evaluation of retinal vascular diseases, including diabetic retinopathy, retinal vein occlusion, uveitis, retinal artery occlusion, and age-related macular degeneration, revealing crucial clinical features such as macular capillary dilation, impaired perfusion, microaneurysms, capillary remodeling, intraretinal fluid, and neovascularization (Spaide and Curcio, 2017). Recently, OCTA has been used in the assessment of systemic diseases such as coronary artery disease, kidney disease, and neurodegeneration disorders (Arnould et al., 2018; Monteiro-Henriques et al., 2022; Shah and Apte, 2020; Vadalà et al., 2019). Several domestic studies have highlighted a significant association between retinal macular vascular density and the severity of CAS, suggesting that OCTA-based detection of retinal microvascular alterations could serve as a non-invasive early indicator for monitoring asymptomatic CAS (Xu et al., 2022). This study aims to utilize OCTA technology to evaluate retinal microcirculation in CAS patients scheduled for carotid angioplasty and stenting. The objectives are to investigate the characteristics of retinal microcirculatory changes in CAS and assessing the predictive value of retinal microcirculation parameters for the therapeutic outcomes and prognosis of CAS patients undergoing surgical treatment.

## Methods

### Study design and participants

This retrospective study was approved by the Ethics Committee of Zhongshan Hospital of Fudan University, and written informed consent was obtained from all patients. A total of 40 eyes from 20 patients with carotid artery stenosis (mean age 65.3 ± 4.5 years) were consecutively enrolled. All patients were diagnosed with >60% diameter stenosis of the internal carotid artery and met the criteria for undergoing unilateral carotid angioplasty artery stenting in the Vascular Surgery Department at Zhongshan Hospital. Comprehensive ophthalmologic examinations were performed both preoperatively and postoperatively. Exclusion criteria were as follows: (1) the presence of other ocular diseases (e.g., glaucoma, retinal vascular diseases) or a history of ocular trauma or intraocular surgery (excluding uncomplicated cataract surgery); (2) significant media opacity affecting imaging quality; (3) diabetic retinopathy; (4) occurrence of CAS-related complications within the past 3 months.

### Surgical treatments

The carotid angioplasty stenting procedure was performed in the Department of Vascular Surgery at Zhongshan Hospital, Fudan University. Patients received dual anti-platelet therapy and statins starting 7 days prior to the procedure. All surgeries were conducted under local anesthesia and were completed via femoral artery puncture following standard protocols. Comprehensive neurovascular angiography was carried out on the aortic arch and its branches, including the bilateral carotid arteries, bilateral subclavian arteries, and the brachiocephalic trunk. Before stent implantation, a distal embolic protection device (EPD) was deployed at the siphon segment, balloon dilation was then performed, followed by the placement of a self-expanding stent at the lesion site. The stent diameter exceeded the lumen of the common carotid artery (for lesions involving both the common and internal carotid arteries) or the internal carotid artery (for lesions confined to the internal carotid artery) by 1–2 mm. The stent length was sufficient to fully cover the lesion. Mannitol solution was administered as needed, and blood pressure was strictly controlled to prevent post-procedure hyperperfusion syndrome. After angiography confirmed significant improvement in the stenotic lesion, the cerebral protection device was removed. Postoperatively, antiplatelet therapy was continued for at least 4 weeks.

### Acquisition of OCTA images

For patients without contraindications to mydriasis, pupil dilation was performed. Once adequate dilation was achieved, imaging of the macular, optic disk, and ultra-wide-angle retina was conducted using a 400 kHz ultra-widefield swept-source optical coherence tomography angiography (UWF SS-OCTA) device (BM-400 K BMizar, TowardPi Medical Technology, Beijing, China). Vessel density was assessed using both the vascular retinal mode (6 × 6 mm, 24 × 20 mm) and the vascular disk mode (6 × 6 mm). The built-in software automatically segmented the retinal vascular layers: the superficial vascular complex (SVC) in the macular region was defined as the layer spanning from the inner limiting membrane (ILM) to the outer boundary of the inner plexiform layer (IPL), while the deep vascular complex (DVC) was defined as the layer between the IPL and the outer plexiform layer (OPL). Peripapillary retinal nerve fiber layer (RNFL) thickness and radial peripapillary capillary (RPC) plexus density were measured and analyzed using optic disk OCTA images. The system also captured the choriocapillaris layer and the mid-to-large choroidal vessel layers. Following layer segmentation, the software automatically extracted and analyzed blood flow density and thickness data in both the optic disk and macular regions, calculated the FAZ area and the foveal density within a 300 μm wide ring surrounding the FAZ (FD-300). Scans with a quality score below 6 or those with significant artifacts detected by an experienced ophthalmologist were repeated to ensure accuracy.

### Statistical analysis

Statistical analysis was performed using SPSS (version 29.0; IBM-SPSS Inc., Chicago, IL). Wilcoxon signed-rank test was used to compare the retinal SVC, DVC, RPC vessel density and other related parameters before and after stent implantation. All measurements are presented as mean ± standard deviation. A two-tailed *p*-value of <0.05 was considered statistically significant.

## Results

From March 2023 to September 2024, OCTA examinations were conducted on 37 patients with carotid artery stenosis. Among them, eight patients with incomplete OCTA images before and after carotid angioplasty and stenting, three patients with diabetic retinopathy, five patients with significant refractive media opacity, and one patient with postoperative complications were excluded. The remaining 20 patients were included as study subjects. Table 1 provides a summary of patient demographics, the degree of carotid artery stenosis, and systemic condition. The severity of carotid artery stenosis ranged from 60 to 99%, with 12 patients (stenosis >90%) classified as having severe stenosis. Each patient underwent two OCTA examinations, 1 day before surgery and the other within 1 week after surgery.

**Table 1:** Characteristics of the study group.

Variables	Subjects (*n* = 20)
Age (year)	65.3 ± 4.5
Sex (male/female)	18/2
Surgery side (R/L)	10/10
Carotid stenosis %	60–99
Diabetes mellitus	3
Hypertension	12
Smoking	7
Cerebral infarction	6
Blood Pressure(mmHg)	145/90 ± 12/8
Fasting Glucose(mmol/L)	8.2 ± 2.3

As shown in Table 2, the study found no statistically significant changes in retinal thickness (RT), choroidal thickness (CT), or RNFL thickness before and after surgery. In the ipsilateral eye group, there was a significant increase in RPC vessel density (46.99 ± 2.68 vs. 46.40 ± 2.77, *p* = 0.028) compared to pre-surgery levels. The superficial retinal microcirculation analysis of OCTA showed that FAZ area, FD-300, and SVC did not show statistically significant differences compared with preoperative, while the deep retinal vascular parameters analysis of OCTA showed that FAZ area (0.62 ± 0.05 vs. 0.53 ± 0.03, *p* = 0.018) was significantly decreased compared with preoperative. FD-300 (44.94 ± 3.29% vs. 42.33 ± 4.08%, *p* = 0.031) and DVC (47.17 ± 3.29% vs. 54.44 ± 3.53%, *p* = 0.004) were significantly higher than those before surgery. However, in the contralateral eye group, the results of OCTA analysis indicated a slight increase in RPC (47.47 ± 2.04% vs. 46.69 ± 2.28%, *p* = 0.112), DVC (46.22 ± 3.63% vs. 45.22 ± 4.51%, *p* = 0.076), and FD-300 (44.54 ± 3.89% vs. 43.96 ± 3.96%, *p* = 0.472) compared with the preoperative values, but it was not statistically significant.

**Table 2:** Structural and vessels densities OCTA analysis in the retinal vascular plexuses before and after carotid angioplasty and stenting in the ipsilateral and contralateral eye groups.

Variables	Ipsilateral eyes			Contralateral eyes		
	Pre-stenting	Post-stenting	p*	Pre-stenting	Post-stenting	p*
RT ( μ m)	360.61 ± 14.74	359.17 ± 16.70	0.288	358.56 ± 15.34	357.78 ± 15.92	0.098
CT ( μ m)	276.39 ± 71.68	262.94 ± 69.45	0.670	296.72 ± 75.57	290.17 ± 74.80	0.289
RNFL thickness ( μ m)	99.33 ± 13.99	101.22 ± 16.01	0.203	101.33 ± 10.79	101.42 ± 9.44	0.938
RPC	46.40 ± 2.77	46.99 ± 2.68	**0.028**	46.99 ± 2.28	47.47 ± 2.04	0.112
OCT-A superficial						
FAZA (mm^2^)	0.19 ± 0.01	0.23 ± 0.01	0.208	0.18 ± 0.01	0.20 ± 0.01	0.263
FD-300(%)	39.56 ± 5.62	41.30 ± 4.47	0.231	40.45 ± 5.69	41.44 ± 5.10	0.500
SVC-VD	43.56 ± 3.50	44.61 ± 3.29	0.546	43 ± 3.20	43.61 ± 3.43	0.245
OCT-A deep						
FAZA (mm^2^)	0.62 ± 0.05	0.53 ± 0.03	**0.018**	0.53 ± 0.05	0.56 ± 0.05	0.777
FD-300(%)	42.33 ± 4.08	44.94 ± 3.29	**0.031**	43.96 ± 3.96	44.54 ± 3.89	0.472
DVC-VD	44.44 ± 3.53	47.17 ± 3.38	**0.004**	45.22 ± 4.51	46.22 ± 3.63	0.076

Bold: statistically significant differences between the two groups.

**Figure 1:**
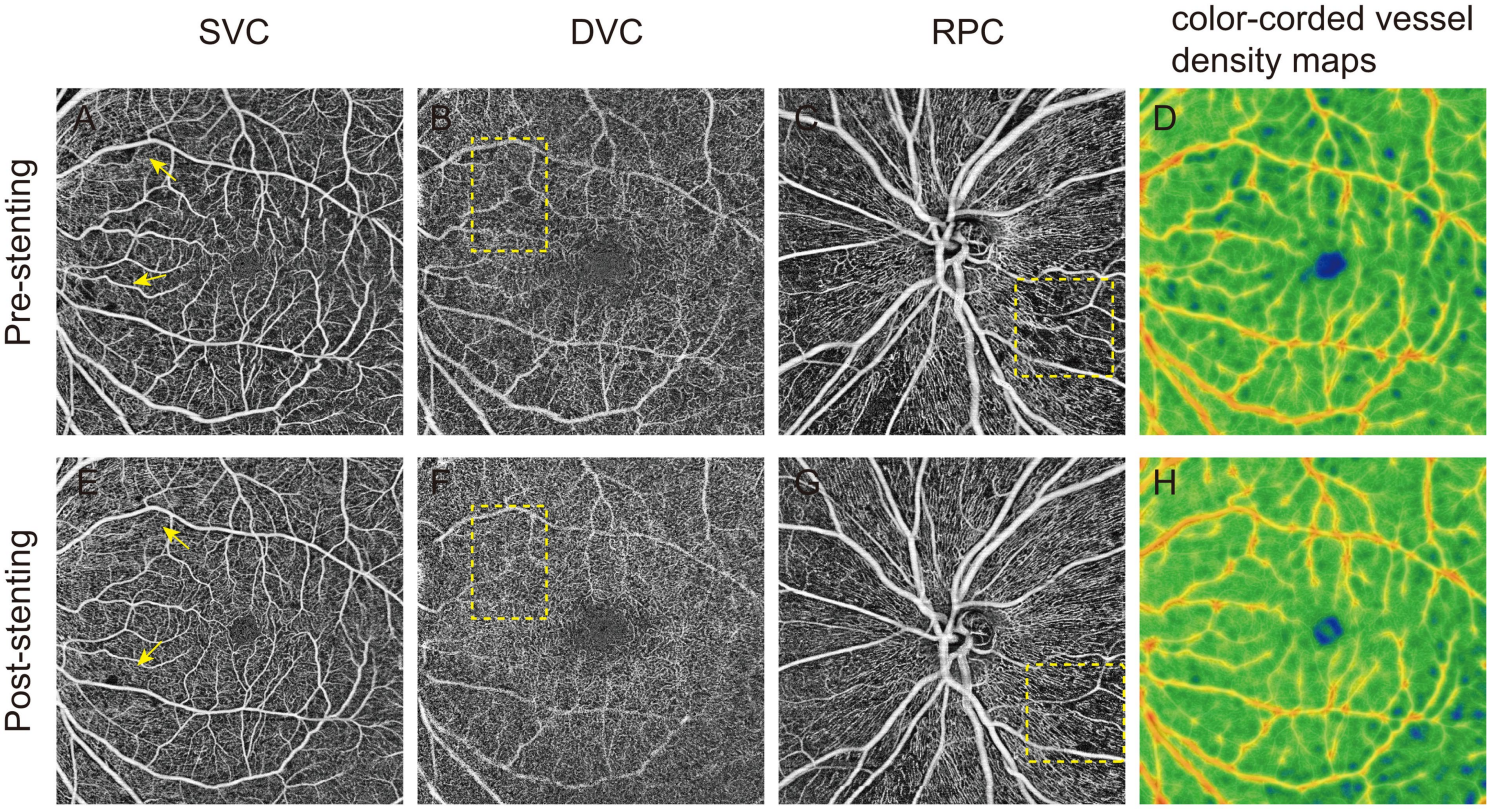
Optical coherence tomography angiography (OCTA) images of patients with carotid artery stenosis (CAS). Preoperative OCTA qualitatively showed the vessel density of superficial vascular complex (SVC), deep vascular complex (DVC), and radial peripapillary capillaries (RPC) and color-coded vessel density map in panel **(A–D)**. After carotid angioplasty stenting, the qualitative images of the 4 regions **(E–H)** of OCTA were significantly improved (yellow square).

**Figure 2:**
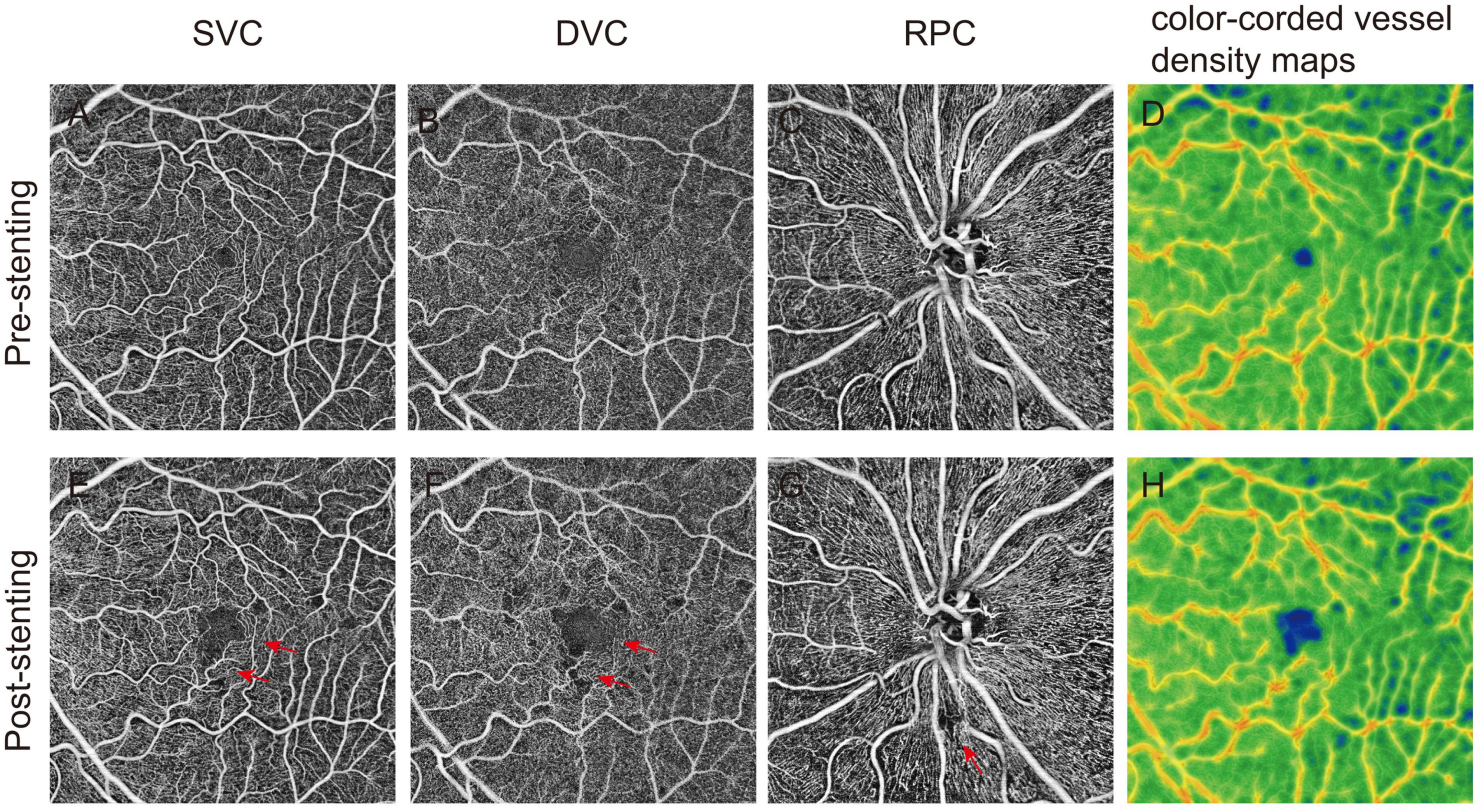
The effects of microembolism on retinal vascular plexus perfusion measured by optical coherence tomography angiography (OCTA). **A-D** showed the vascular density of superficial vascular complex (SVC), deep vascular complex (DVC), radial peripapillary capillaries (RPC) and color-coded vessel density map recorded by OCTA before operation. The postoperative **(E-H)** images of patients with microembolization showed a significant decrease in vascular density. En-face OCTA images demonstrated an increased area of flow void in the corresponding vascular territories (red arrowhead).

The findings suggest that carotid artery angioplasty and stenting improves retinal microvascular circulation in the ipsilateral eye, primarily reflected by increased blood flow density in the deep retinal vessels and RPC, as shown in Figure 1. In addition, in one of the observed cases, there was no abnormality before surgery, but 3 days postoperatively, the patient developed blurred vision, accompanied by poor movement of the right upper limb, nausea and vomiting. OCTA image analysis demonstrated a significant increase in the FAZ area and retinal non-perfusion regions, accompanied by a marked decrease in blood flow density (Figure 2), suggesting the occurrence of retinal ischemia. Surgical records for this patient indicated that a carotid artery shunt was not utilized during the procedure, raising the possibility of intracranial hyperperfusion or microembolism after the surgery. These observations highlight the potential of OCTA as a non-invasive and sensitive tool for quantitatively assessing retinal blood flow changes. Such assessments can effectively evaluate surgical efficacy and provide early indications of potential adverse outcomes, offering critical feedback for the management of carotid artery stenosis surgery.

**Figure 3:**
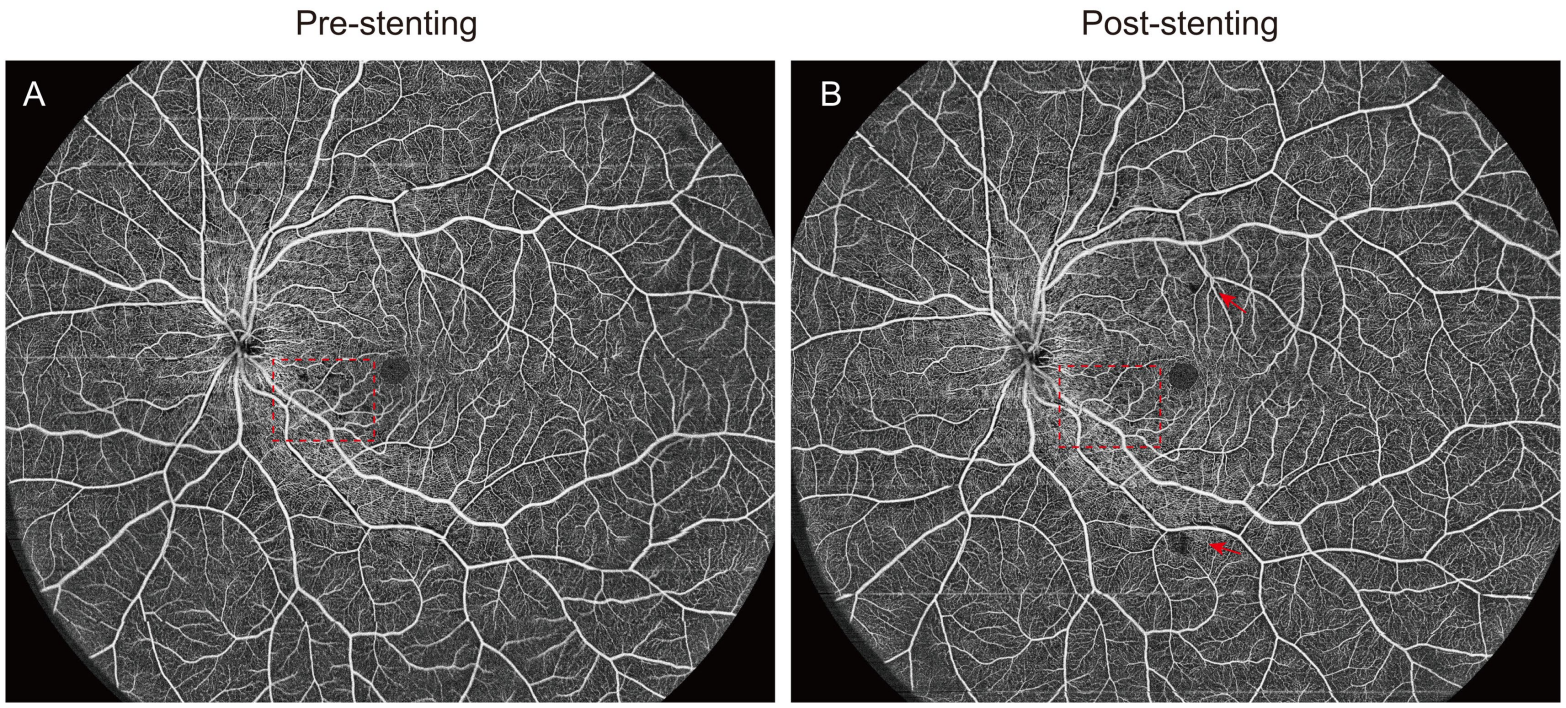
The ultra-wide field OCTA images of CAS patients pre- and post-stenting. **(A,B)** The red square showed the improvement of vessel density in the macular parafovea before and after surgery,and the red arrow indicated the new non-perfusion area after surgery.

For 10 patients with higher compliance, an ultra-wide field 24 × 20 mm OCTA scan was performed to obtain more comprehensive retinal blood flow data. This study is the first to quantify the non-perfusion area in 24 × 20 mm image as a metric to evaluate changes in retinal blood supply following carotid artery stenosis surgery. Data analysis revealed a significant reduction in the non-perfusion area within the deep retinal vascular plexus of the ipsilateral eye after stenting (44.24 ± 21.78 vs. 63.26 ± 19.02, *p* = 0.028), while no statistically significant change was observed in the contralateral eyes (Table 3). As shown in Figure 3, UWF-OCTA enface images clearly captured the changes in the non-perfusion area of blood flow around the optic disk and parafovea of the macula, indicating that the changes in blood flow density in the macula and optic nerve region are more sensitive, which is consistent with previous research results (Pierro et al., 2021). Interestingly, post-operative OCTA image analysis also revealed changes in the perivascular arch non-perfusion area. These results suggest that short-term alterations in retinal microcirculation following CAS surgery not only affect macular and peripapillary blood flow density but also impact blood perfusion in the vascular arch and peripheral retina. Due to the difficulty of obtaining complete and high-quality UWF OCTA images, only a limited number of cases were included in the statistical analysis of the non-perfusion area, limiting the statistical credibility, though the findings retain some reference value.

**Table 3:** The ultra-wide field OCTA analysis in the no perfusion area before and after carotid angioplasty and stenting in the ipsilateral and contralateral eye groups.

*N* = 13	Ipsilateral eyes			Contralateral eyes		
	Pre-stenting	Post-stenting	p*	Pre-stenting	Post-stenting	p*
No perfusion area (mm^2^)						
Superficial	42.05 ± 20.31	25.66 ± 16.35	0.063	26.62 ± 18.02	26.53 ± 13.83	0.889
Deep	63.26 ± 19.02	44.24 ± 21.78	**0.028**	47.80 ± 17.69	51.34 ± 25.87	0.779

Bold: statistically significant differences.

## Discussion

CAS as a common clinical condition, can lead to ocular ischemic lesions that affect the retinal and optic nerve blood supply. Previous studies have reported significantly reduced retinal blood flow density in CAS patients compared to healthy controls, particularly in those with moderate to severe CAS, where the macular and optic nerve blood flow densities are significant impaired, indicating compromised retinal vascular function (Hou et al., 2024; Pierro et al., 2021). Research suggests that when carotid artery stenosis exceeds 50%, the ocular blood supply is notable reduced (Liu et al., 2022). In cases of severe stenosis (>90%), the reduction in retinal blood flow may result in pronounced ischemic damage.

In this study, OCTA technology was used to assess and analyze short-term retinal microcirculatory changes in CAS patients before and after carotid stenting. The results revealed that in the ipsilateral eye group, there was no significant change in the parameters related to macular superficial vessels. However, in the parameter analysis of the deep vascular layer, statistically significant changes were observed in DVC, FAZA, FD-300 and RPC-VD. In contrast, no statistically significant changes in vessel density were observed in either the superficial or deep layers of the contralateral eye. These findings are in part consistent with previous studies that reported a significant increase in DVC vessel density in the ipsilateral eye after stent implantation (Lee et al., 2019); The same changes were observed in the contralateral eye. Therefore, they suggested that unilateral treatment of severe CAS may increase macular vascular density in both eyes. However, it is clear that our findings do not support such a conclusion.

The FAZ area (FAZA) and FD-300 are important indicators for evaluating retinal blood supply status (Drinkwater et al., 2020). Enlargement of the FAZA is an early sign of retinal ischemic damage and can be quantitatively assessed using OCTA for monitoring disease progression. Similarly, FD-300 serves as a useful parameter for indicating macular ischemia. However, FAZA parameters can be influenced by the selection of flow signal layers and retinal imaging area. This study standardized FAZ analysis across different macular layers within a 6 × 6 mm scanning range and observed significant improvements in FAZA and FD-300 in the deep vascular layer of the ipsilateral eye after stenting. The RPC consists of a unique capillary network within the radial peripapillary nerve fiber layer, characterized by long, straight capillaries aligned parallel to nerve fiber bundles, with few anastomoses (Yu et al., 2014). Previous research has found a significant decrease in RPC-VD in CAS patients, reflecting reduced optic disk vascular supply and the potential for retinal ganglion cell apoptosis (Lahme et al., 2018). In this study, RPC-VD showed slight improvement following carotid stenting.

This study is the first to conduct 24 × 20 mm UWF OCTA imaging in CAS patients to comprehensively evaluate retinal perfusion in both the posterior pole and peripheral areas. The analysis revealed a significant reduction in non-perfusion areas within the deep retinal vascular layer in the macular region and around the optic disk on the ipsilateral side following carotid angioplasty and stenting. However, an increase in non-perfusion areas was observed near the vascular arcades, suggesting that peripheral retinal blood supply undergoes noticeable changes after the resolution of carotid artery stenosis. Interestingly, these changes in the peripheral retina may contrast with the trends observed in macular retinal blood flow density.

Previous studies have suggested that in patients with severe unilateral carotid stenosis, stenting can improve macular vessel density in both eyes (Lee et al., 2019; Machalińska et al., 2017). This is thought to occur due to reduced reliance on collateral circulation in the contralateral hemisphere, leading to blood flow redistribution and improved bilateral retinal perfusion. However, this study did not observe statistically significant changes in contralateral eye blood flow density, potentially due to the small sample size and variability in the degree of carotid stenosis among patients. Nonetheless, the comparison of pre- and post-stenting blood flow density indicates that carotid stenting can significantly increase DVC blood flow density, reflecting improvements in retinal microcirculation.

This study has several limitations that should be acknowledged. The small sample size, particularly for UWF OCTA imaging, may limit the statistical power and generalizability of the findings. Additionally, the short-term postoperative observation period does not provide insights into the long-term effects of carotid artery stenting on retinal microcirculation. Variability in patient characteristics, such as the degree of stenosis and systemic comorbidities, may also introduce confounding factors. Moreover, while OCTA provides high-resolution imaging, artifacts, especially in UWF imaging, could affect the accuracy of quantitative assessments, such as non-perfusion area measurements.

Future research should include larger and more diverse patient cohorts and adopt longitudinal designs to evaluate the long-term impact of carotid stenting on retinal vascular health. Combining OCTA with other diagnostic tools, such as Doppler ultrasound, neuroimaging techniques or functional retinal assessments, could provide a more comprehensive understanding of carotid interventions. Advances in imaging technology, including enhanced artifact correction and wider scanning fields, may further improve the precision of retinal microcirculation evaluation. These efforts could establish standardized retinal biomarkers as non-invasive tools for monitoring vascular diseases, guiding treatment strategies, and predicting patient prognosis.

## Conclusion

This study highlights the role of carotid artery angioplasty and stenting in restoring retinal microcirculation, particularly in the deep retinal vascular plexus of the ipsilateral eye. Using OCTA and ultra-wide-field imaging, we identified both improvements in macular perfusion and distinct changes in peripheral retinal blood flow. These findings underscore the potential of OCTA-derived parameters as objective markers for evaluating the vascular impact of carotid interventions. Future research should further refine these insights to enhance the management and prognosis of CAS-related vascular and retinal complications.

## Data Availability

The raw data supporting the conclusions of this article will be made available by the authors, without undue reservation.
